# Case of Ischuria Renalis, in Which the Secretion of Urine Was Suspended for Several Days—Recovery of the Patient

**Published:** 1847-05

**Authors:** 


*Case of Ischuria Renalis, in which the secretion of urine was suspended for several days—Recovery of the Patient.*

The following remarkable case of this most dangerous disease occurred in our practice several years ago, and is presented with a view of showing to what extent the malady may progress and yet terminate favorably. The patient, a mulatto girl, about eighteen years of age, was visited for the first time, on the 11th day of Nov. 1843. She complained of pain in the head and back, and constipated bowels ; her pulse was full and strong, and the temperature of the skin very much increased. She was bled to the amount of twelve or fourteen ounces, and an active cathartic ordered. For the two subsequent days she was entirely free from any symptoms of disease, but on the third she complained of vertigo and excessive pain in the lumbar region, and for the first time disclosed the scanty secretion of urine, not more than a half-gill being secreted in the twenty-four hours. Towards evening the sensorium seemed affected; the patient being drowsy evinced an indisposition to exertion of any kind; the eyes yellow and suffused; pulse slow and full; with a slight tendency to stiffness in the limbs. The catheter was introduced into the bladder, but no urine was discharged. The symptoms indicating the abstraction of blood, eighteen ounces were drawn from the arm, a murcurial cathartic administered, and a blister applied to the lumbar region. *From this date, the 14th, until the 24th, not a drop of urine passed from the bladder*, although the catheter was introduced daily. During the whole of this time, the sensorium was more or less affected; the patient at one period was delirious, and again apparently comatose. On the 16th, she was attacked with stiffness of the limbs, which increased until the fore-arm became flexed, the fingers closed on the palms, and the legs spasmodically extended, when the paroxysm would gradually disappear after a continuance of some two hours. The paroxysms recurred at irregular intervals for about three weeks, several days after the secretion of urine was restored.—During a paroxysm the whole muscular system seemed to be affected—the face flushed—the pulse full and hard—the skin hot and bathed in profuse perspiration; but towards its termination, an opposite state of things would prevail, and the patient awake up, completely prostrated in mind and body. The high state of excitement under which she labored, induced me to resort to venesection, the warm bath, &c., but without effect; in fact, all remedies used appeared rather to increase than diminish the violence of a paroxysm. At last, an unusually severe paroxysm having occurred on the 24th, recourse was had to an enema of tobacco, made by infusing two drachms of tobacco in a pint of boiling water. In a very few minutes after its administration the patient became intensely sick, and made violent efforts to vomit, while the rigidity of the muscular system speedily disappeared. It became necessary to repeat the injection daily as long as the spasms continued. Immediately after the close of the paroxysm on the morning of the 24th the patient complained of fulness in the supra-pubic region; the catheter was introduced into the bladder and six ounces of pure pus without any admixture of urine, were discharged. In the evening, about two ounces more were drawn mixed with urine—the first she had passed since the 14th, a period of eleven days. From this time her improvement was manifest, the spasms becoming less frequent and violent, and the urine increasing in quantity, until about the middle of December, when she was discharged as cured.

The foregoing case is certainly remarkable for its duration ; as very few cases of this sure and usually fatal disease are extended beyond the eight or ninth day, death most commonly terminating the patient's suffering even before that period. So far as we are aware, the period of time between the cessation and the reappearance of the secretion of urine, is longer than of any recorded case, in which recovery took place.

The administration of tobacco for the relief of spasm of an hysterical and tetanic nature, although of ancient origin, is not, we believe, general with the profession. Its use in the present instance was of undoubted utility, as was evinced by its power in controlling the paroxysms, after other remedies had failed, and in several instances since we have observed its good effects. The cases in which we have exhibited the tobacco, were such as were possessed of a vigorous constitution, with a full, tense pulse, and in short all the evidences of high arterial excitement. To such, and such alone, is the remedy applicable. Cases of an opposite description we need not say would be injured instead of benefitted by its administration.—*Robert E. Little, M. D. in Southern Med. and Surg. Journal.*

